# Age-Related Changes in the Glycolytic Enzymes of M2-Isoform of Pyruvate Kinase and Fructose-1,6-Bisphosphate Aldolase: Implications to Age-Related Macular Degeneration

**DOI:** 10.14336/AD.2024.0077

**Published:** 2024-10-01

**Authors:** Ammaji Rajala, Raju V. S. Rajala

**Affiliations:** ^1^Department of Ophthalmology; ^2^Department of Biochemistry and Physiology, and; ^3^Department of Cell Biology, University of Oklahoma Health Sciences Center; ^4^Dean McGee Eye Institute, Oklahoma, Oklahoma 73104, USA.

**Keywords:** Aerobic glycolysis, pyruvate kinase, aldolase, age-related macular degeneration, retina, retinal pigment epithelium

## Abstract

Prior studies have emphasized a bioenergetic crisis in the retinal pigment epithelium (RPE) as a critical factor in the development of age-related macular degeneration (AMD). The isoforms Fructose-1,6-bisphosphate aldolase C (ALDOC) and pyruvate kinase M2 (PKM2) have been proposed to play a role in AMD pathogenesis. While PKM2 and ALDOC are crucial for aerobic glycolysis in the neural retina, they are not as essential for the RPE. In this study, we examined the expression and activity of PKM2 and ALDOC in both young and aged RPE cells, as well as in the retina and RPE tissue of mice, including an experimentally induced AMD mouse model. Our findings reveal an upregulation in PKM2 and ALDOC expression, accompanied by increased pyruvate kinase activity, in the aged and AMD mouse RPE. Conversely, there is a decrease in ALDOC expression but an increase in PKM2 expression and pyruvate kinase activity in the aged and AMD retina. Overall, our study indicates that aged and AMD RPE cells tend to favor aerobic glycolysis, while this tendency is diminished in the aged and AMD retina. These results underscore the significance of targeting PKM2 and ALDOC in the RPE as a promising therapeutic approach to address the bioenergetic crisis and prevent vision loss in AMD.

## INTRODUCTION

The energy processes in the retina are crucial for its health [1-4]. In a healthy retina, glucose from the choroid is efficiently used by photoreceptors through glycolysis, producing ATP and lactate [5]. The retinal pigment epithelium (RPE) processes this lactate by converting it to pyruvate and using it in oxidative phosphorylation (OXPHOS) [5]. Additionally, lipids from phagocytosed outer segments undergo β-oxidation in mitochondria, contributing to ATP generation [6].

In age-related macular degeneration (AMD), damaged mitochondria in the RPE lose their ability to generate ATP, leading to increased reliance on glycolysis [6]. This altered metabolic state reduces the availability of glucose to photoreceptors, resulting in a decrease in lactate supplied to the RPE. The disruption in the normal energy balance causes a cell-specific shift, ultimately disturbing the metabolic ecosystem [5]. This disturbance contributes to the decline of both photoreceptors and the RPE, suggesting that metabolic uncoupling may be the central issue in AMD [6]. It underscores the critical role of retinal energy processes in maintaining retinal health.

A prior investigation revealed that sera from patients with AMD react with the M2 isoform of retinal pyruvate kinase (PKM2) and the C isoform of aldolase (ALDOC) [7]. The presence of PKM2 IgG is correlated with the stage of AMD [7]. Preclinical studies indicated a decrease in ALDOC and PKM2 expressions in the retinas of aged mice, with an increased deposit of PKM2 in the aged RPE [7]. In contrast to the PKM1 isoform, PKM2 is allosterically activated by fructose 1,6-bisphosphate (FBP), promoting OXPHOS [8]. The phosphorylated form of PKM2 is unable to bind FBP, thereby promoting aerobic glycolysis or the Warburg effect [9]. The primary way neurons metabolize glucose is through aerobic glycolysis, which safeguards against oxidative damage [10]. When PKM2 is deleted in mice, there is a shift from aerobic glycolysis to OXPHOS in neuronal somata [10]. This transition results in oxidative damage and the gradual decline of dopaminergic neurons [10]. Fructose-bisphosphate aldolase, commonly referred to as aldolase, is a glycolytic enzyme serving as a glucose sensor [11]. It regulates the concentration of FBP in cells by cleaving FBP into triose phosphates, dihydroxyacetone phosphate (DHAP), and glyceraldehyde 3-phosphate (G3P) [12]. Both of these 3-carbon sugars are crucial for glycerol synthesis, which forms the backbone of lipids [13]. Aldolase exists in three isoforms: A, B, and C (17), with Aldolase C expressed in the retina and RPE [14]. However, there are currently no detailed studies available on PKM2 and ALDOC during aging in the retina and RPE.

This study aims to investigate the expression and activities of PKM2 and ALDOC in young and aged retinas, as well as in a well-established experimental mouse model of AMD. Our findings suggest that the activities of these two enzymes are altered in aging and AMD in both the retina/photoreceptors and RPE.

## MATERIALS AND METHODS

### Antibodies

Polyclonal PKM1 (Cat# 7067), PKM2 (Cat # 4053), and pPKM2-Y105 (Cat# 3827) antibodies were acquired from Cell Signaling (Danvers, MA). A monoclonal actin (Cat# MA1-744) antibody was purchased from ThermoFisher Scientific (Dallas, TX). Monoclonal aldolase C (Cat# MCA-4A9) antibody was obtained from EnCor Biotechnology Inc (Gainesville Florida). Polyclonal LDHA (Cat# 21799-1-AP) and LDHB (Cat# 14824-1-AP) antibodies were obtained from Proteintech (Rosemont, IL). Dr. James F. McGinnis (University of Oklahoma Health Sciences Center) provided the monoclonal 1D4 rhodopsin antibody and Dr. Jian-Xing Ma (University of Oklahoma Health Sciences Center) provided the polyclonal Rpe65 antibody. The monoclonal arrestin antibody was a kind gift from Dr. Paul Hargrave (University of Florida).

### Chemicals

All other reagents were of analytical grade and purchased from Sigma (St. Louis, MO).

### Animals

Animal experiments were conducted as per the guidelines of the *Association for Research in Vision and Ophthalmology Statement for the Use of Animals in Ophthalmic and Vision Research* and the *NIH Guide for the Care and Use of Laboratory Animals.* The protocols were approved by the IACUC at the University of Oklahoma Health Sciences Center. Animal breeding was carried out in the DMEI vivarium. All animals were raised under dim cyclic light (40-60 lux, 12 h dark/light cycle). Retinas were immediately removed after euthanasia and were frozen in liquid nitrogen. Eye tissues were harvested for biochemistry or immunohistochemistry.

### Pyruvate kinase enzyme assay

The enzyme activity of pyruvate kinase (PK) was assessed using the lactate dehydrogenase (LDH) coupled enzyme assay [15]. The assay was conducted in the presence of a mouse retinal lysate that contained an enzyme buffer mixture, comprising 50 mM Tris-HCl (pH 7.4), 100 mM KCl, 5 mM MgCl2, 1 mM ADP, 0.5 mM PEP, and 0.2 mM NADH (reduced form of NAD+) and 8 U of LDH. The spectrophotometric measurement of PK activity was performed by monitoring the reduction in absorbance at 340 nm resulting from the oxidation of NADH.

### Aldolase enzyme assay

The assay was conducted following the previously described method (16). The reaction mixture consisted of 0.1M potassium acetate buffer at pH 8.0, 0.1-5 mM FBP, 0.2 mM NADH, and a combination of coupling enzymes, namely glycerol phosphate dehydrogenase and triosephosphate isomerase. The reaction was initiated by introducing retina or RPE lysate and monitoring the reduction in absorbance at 340 nm due to the oxidation of NADH.

### Sodium iodate injection

Sodium iodate (NaIO_3_) was diluted in PBS at a concentration of 2.5 mg/ml and injected through the tail vein to C57Bl/6 mice at a concentration of 20 mg/kg body weight [16]. Control mice were injected with PBS instead of sodium iodate. After treatment, mice were euthanized on day 3, and examined the RPE and retina morphology and immunohistochemistry.

### ARPE-19 cell culture

ARPE-19 cells are of human origin. We grew them in an F12 medium containing 10% fetal bovine serum (FBS), 2 mM L-glutamine, 0.1 mg/ml streptomycin, and 100 U/ml penicillin (Gibco). Long-term cultures (4 months) were not passaged but replaced medium twice per week.

### Other Methods

Paraffin sections were prepared using prefer as fixative for immunohistochemistry, and immunoblot analysis was carried out according to the method we published previously (6). We have isolated RPE without choroid/sclera contamination as described earlier [17]. For all immunofluorescence staining experiments, ARPE-19 cells, RPE, and retina sections from the same batch were stained with identical antibodies (both control and experimental) and imaged simultaneously using consistent microscope settings. Additionally, retina and RPE sections were stained solely with secondary antibodies as negative controls to assess the specificity of primary antibodies and determine background noise levels for image acquisition. The images presented are representative of samples obtained from three retinas/RPEs each from young and aged mice. These images depict the same section viewed under filters to detect green and red fluorescence, with DAPI-stained nuclei appearing blue.

### Statistical Analysis

We employed GraphPad Prism 9 software for statistical analysis. All collected samples were included in the analysis, and various statistical methods were applied based on the experiment type. Before selecting the appropriate statistical analysis, we conducted normality tests (Anderson-Darling, D’Agostino & Pearson, Shapiro-Wilk, Kolmogorov-Smirnov) to assess the distribution of the data—whether it followed a Gaussian or non-Gaussian pattern. If the data were not normally distributed, we utilized unpaired non-parametric tests for group comparisons, specifically employing the Mann-Whitney U test. The resulting p values were used for inference.

## RESULTS

### Glycolytic enzyme expression in ARPE-19 RPE cells

**Figure 1.**
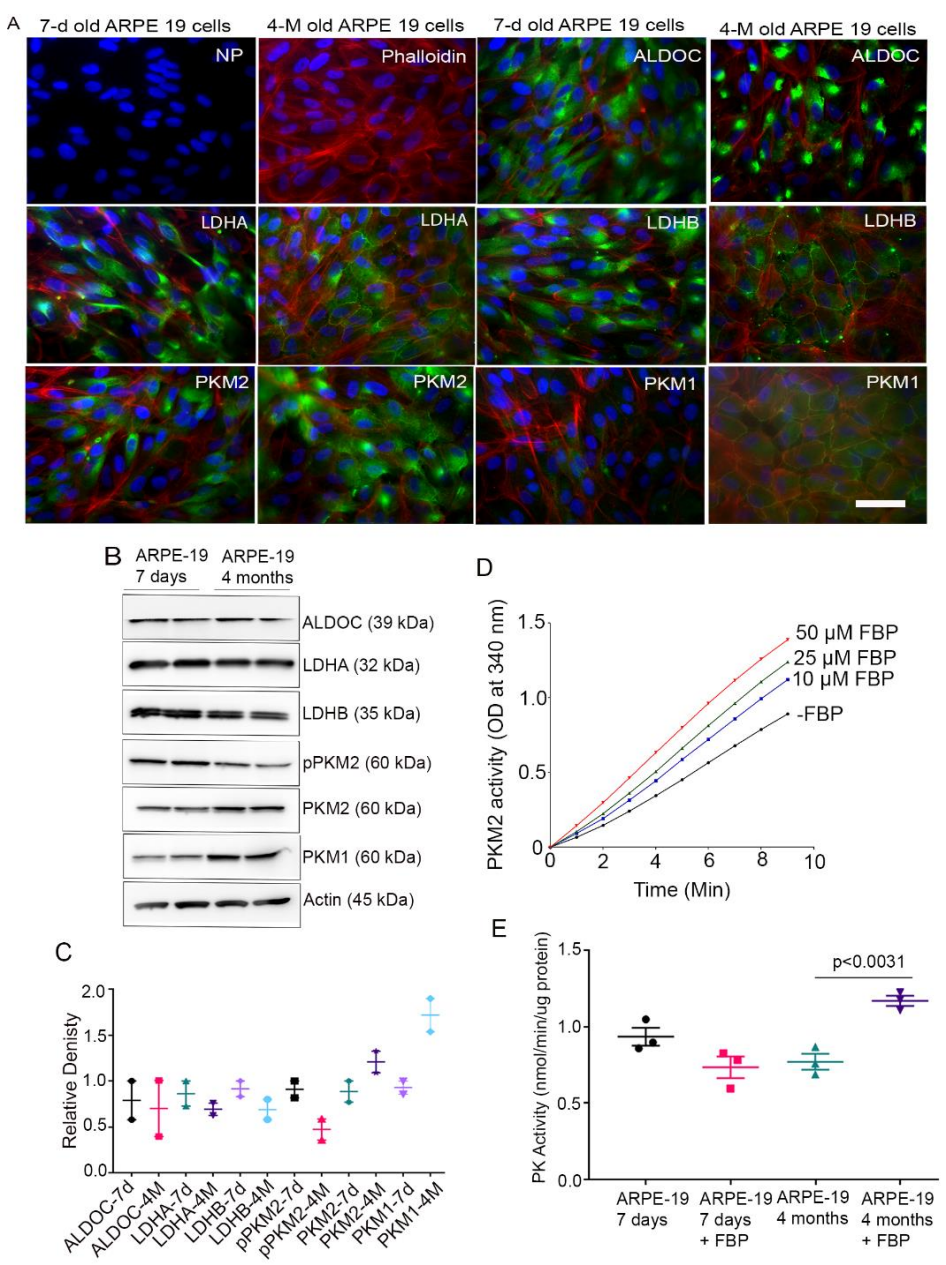
Expression of Glycolytic Enzymes in Young and Aged ARPE19 Cells. Immunocytochemistry was performed on 7-day-old ARPE-19 cells, and 4-month-old ARPE-19 cells (A), utilizing antibodies against ALDOC, LDHA, LDHB, PKM1, and PKM2. Scale bar = 50 µm. Immunoblot analysis of ARPE19 cell protein samples was conducted, probing for ALDOC, LDHA, LDHB, pPKM2, PKM1, PKM2, and actin (B). Densitometric analysis of the tested proteins was normalized to actin, demonstrating the relative protein expression levels (C). Data are mean + *SEM, (n=2).* The pyruvate kinase activity of purified PKM2 was measured using an LDH-coupled enzyme assay. The assay was conducted in the presence of various concentrations of FBP (0-50 µM) along with 0.5 mM PEP (D). The data are presented as mean ± *SEM* (*n=5*). Pyruvate kinase activity was assessed in young and aged ARPE19 cells in the presence and absence of FBP (50 µM) (E). Data are mean + *SEM* (*n*=3). We used the nonparametric Mann-Whitney U test to examine the significance between the two groups.

The ARPE-19 is a human retinal epithelial cell line [18] and is commonly used as an alternative to native RPE cells. Investigators have used the undifferentiated cells as a model for RPE functions; however multiple passages result in the loss of RPE cell phenotype and do not mimic native RPE. Moreover, these cells do not exhibit the native hexagonal shape/cobble-stone morphology of the RPE cell. It has been shown previously that low passage and long-term culture of the ARPE-19 cells to 4 months establishes native RPE cell morphology and shape and expresses RPE-cell specific genes [19]. Seven-day younger and 4-month-old ARPE-19 cells were fixed and subjected to immunocytochemistry with antibodies against ALDOC, lactate dehydrogenase A (LDHA), LDHB, M1 isoform of pyruvate kinase (PKM1), and M2 isoform of pyruvate kinase PKM2) proteins. We co-stained the cells with phalloidin, which stains F-actin around the border of the RPE cell. The younger ARPE-19 cells are undifferentiated and did not exhibit native RPE cell morphology whereas 4-month-old cells show hexagonal native RPE cell morphology (Fig. 1A). The results indicate the expression of ALDOC is observed in both younger and older RPE cells; however, the staining pattern in older cells is distinct from that of younger cells (Fig. 1A). In 7-day-old RPE cells, the ALDOC expression is diffuse and fills the entire cell, whereas in 4-month-old RPE cells, it is concentrated in one region. The significance of this pattern is currently unknown. The LDHA and LDHB expressed in both younger and older cells, and the expression appears to be higher in younger cells compared to older cells (Fig. 1A). The expression of PKM1 appears to be low in younger cells compared to older cells whereas PKM2 expression is lower in younger cells compared to older cells (Fig. 1A). Since immunocytochemistry is not a quantitative measure, we carried out immunoblot analysis with the antibodies used in the immunohistochemistry and normalized their levels to actin (Fig. 1B). The levels of ALDOC, LDHA, and LDHB are comparable between younger and older RPE cells (Fig. 1C). The levels of PKM1 is higher in older RPE cells compared to younger cells whereas the PKM2 levels are higher in older RPE cells compared to younger cells; however, these differences are not statistically significant (Fig. 1C). PKM2 undergoes tyrosine phosphorylation on Y105 [20] and we found decreased phosphorylation of PKM2 with pPKM2 antibody in older RPE cells compared to younger RPE cells, however, this difference is not statistically significant (Fig. 1C). The total pyruvate kinase (PK) activity cannot differentiate the activity from PKM1 or PKM2. Both PKM1 and PKM2 catalyze the conversion of phosphoenolpyruvate (PEP) to pyruvate, but PKM1constitutively converts PEP to pyruvate but PKM2 is allosterically activated by Fructose 1,6-bisphosphate (FBP) [8]. We examined the effect of FBP on PKM2 activity (Fig. 1D). The results indicate that FBP dose-dependently stimulates the activity of purified PKM2 protein *in vitro* (Fig.1D). Then, we carried out PK activity from younger and older RPE cells in the presence of FBP and compared the results to PK activity in the absence of FBP. Our results indicate that there was no effect of FBP on PK activity in younger RPE cells, but the PK activity was significantly higher in older cells in the presence of FBP compared to younger and older RPE cells in the absence of FBP (Fig. 1E). These observations suggest that older RPE cells express PKM2, indirectly indicating that these cells favor glycolysis compared to younger RPE cells. However, the ARPE-19 cell culture system does not fully mimic the *in vivo* situation, as ARPE-19 cells are an immortalized cell line with disrupted metabolic activity. During the immortalization process, cells with specific metabolic advantages are selected, which requires careful consideration when extrapolating the metabolic results to an *in vivo* system.

### Relationship between ALDOC and PKM2 regulation

**Figure 2.**
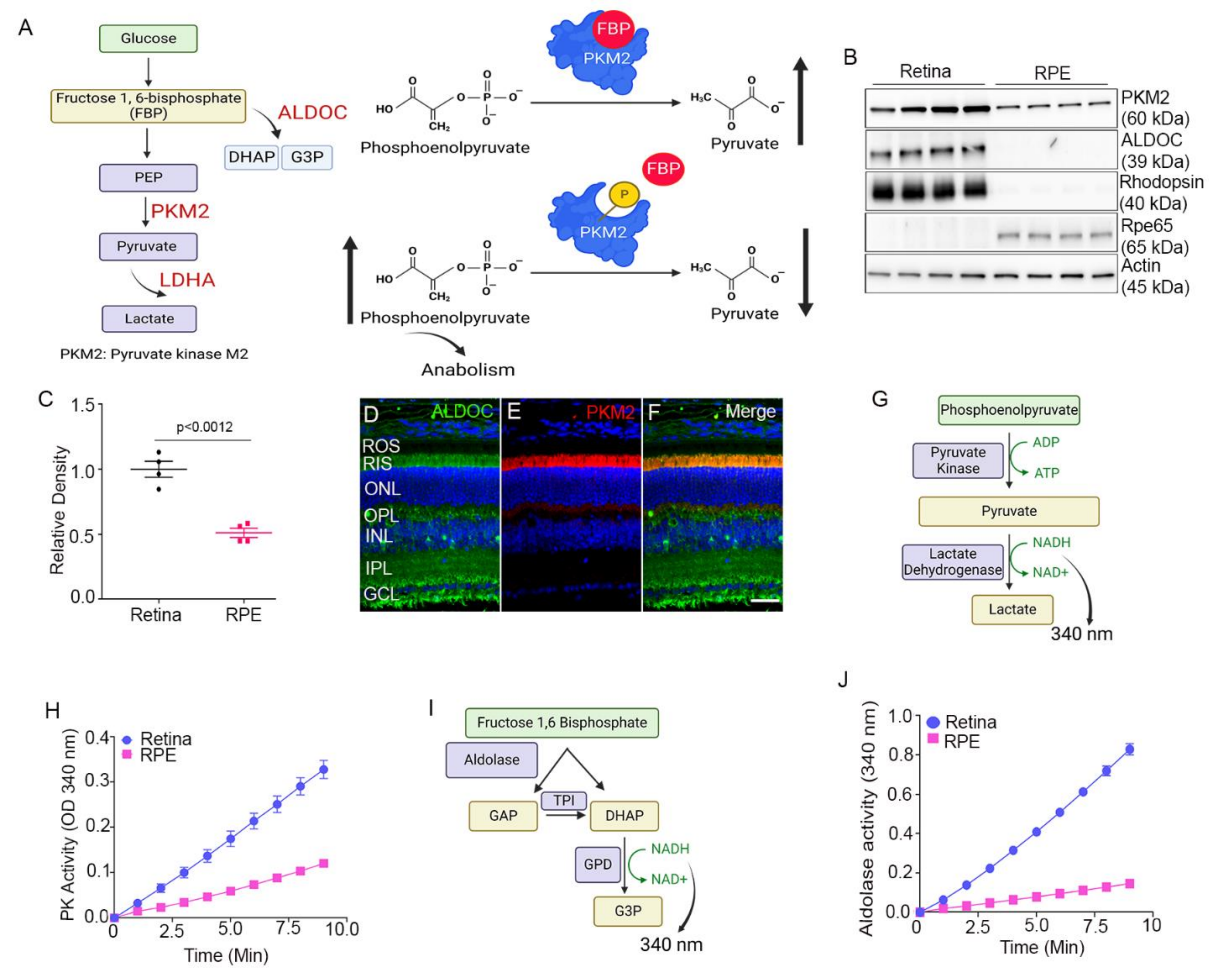
Effect of FBP on PKM2 and Aldolase Activity in RPE and Retina. Schematic representation of the molecular events depicting the impact of FBP on PKM2 activation, tyrosine phosphorylation-induced inactivation, and the subsequent cleavage of FBP by aldolase, leading to the generation of dihydroxyacetone phosphate (DHAP) and glyceraldehyde 3-phosphate (G3P) (A). Immunoblot analysis of retinal and RPE proteins using antibodies against PKM2, ALDOC, rhodopsin, Rpe65, and actin (B). Densitometric analysis of PKM2 expression in the retina and RPE normalized to actin (C). Data (RPE expression relative to the retina) are mean ± *SEM (n=4).* Immunostaining of mouse retina sections for ALDOC (D) and PKM2 (E). Merged image of ALDOC/PKM2 (F). ROS, rod outer segments; RIS, rod inner segments; ONL, outer nuclear layer; OPL, outer plexiform layer; INL, inner nuclear layer; IPL, inner plexiform layer; GCL, ganglion cell layer. Scale bar, 50 μm. Pyruvate kinase activity was measured spectrophotometrically using an LDH-coupled enzyme assay, monitoring the reduction in absorbance at 340 nm from the oxidation of NADH (G) in the retina and RPE (H). Data are mean ± *SEM (n=3).* Aldolase activity was measured in the presence of FBP, NADH, and a mixture of coupling enzymes (glycerol phosphate dehydrogenase - GPD, triosephosphate isomerase - TPI). The reaction was initiated by aldolase addition, monitoring the decrease in absorbance at 340 nm from NADH oxidation (I). Aldolase activity was measured using equal amounts of retina and RPE protein (J). Data are mean ± *SEM, (n=3).* We used the nonparametric Mann-Whitney U test to examine the significance between the two groups.

The allosteric activator fructose 1, 6-bisphosphate (FBP) binds to PKM2 and promotes the conversion of PEP to Pyruvate (Fig.2A). Tyrosine phosphorylation of Y105 in PKM2 causes a steric hindrance for the FBP binding which results in decreased affinity for PEP results in reduced pyruvate generation (Fig. 2A). The FBP concentration in the cell regulates the PEP utilization and generation of pyruvate. The Fructose-1,6-bisphosphate Aldolase (ALDO) catalyzes the reversible aldol reaction of cleavage of FBP to dihydroxyacetone phosphate (DHAP) and glyceraldehyde 3-phosphate (G3P) (Fig. 2A). Aldolase exists as three isozymes, A, B, and C isoforms and are differentially expressed in tissues and are encoded by three different genes [21]. Aldolase C-isoform is predominantly expressed in brain tissues whereas A-isoform is in the brain as well as in muscle and B-isoform expression has been reported in the liver, kidney, and enterocytes [22].

We employed a method for separating proteins from the RPE that is free from the retina and choroidal contamination (20). Immunoblot analysis identified RPE65, an RPE-specific protein, in the RPE lysates, but not in the retina (Fig. 2B). The RPE lysates were devoid of rhodopsin, which is abundant in the retina (Fig. 2B). Immunoblots probed with PKM2 and ALDOC, antibodies showed increased expression of these proteins in the retina compared with the RPE, whereas ALDOC expression was absent from the RPE (Fig. 2B). The expression of PKM2 in the retina is significantly higher than in the RPE. (Fig. 2C). Prefer-fixed retina sections from mice were stained for ALDOC and PKM2 expression (Fig. 2D-F). PKM2 is predominantly expressed in rod inner segments (RIS) and outer plexiform layer (OPL) (Fig. 2E), whereas ALDOC expression can be seen in RIS, OPL, and inner plexiform and ganglion cell layers of the retina (Fig. 2D). The ALDOC is colocalized with PKM2 in the inner segment (Fig. 2F).

The lactate dehydrogenase (LDH) coupled enzyme assay was used to measure pyruvate kinase (PK) enzyme activity [15]. The PK activity was measured spectrophotometrically by monitoring the reduction in the absorbance at 340 nm from the oxidation of NADH (Fig. 2G). Equivalent amounts of retina and RPE proteins were examined for PK activity and the results indicate increased PK activity in the retina compared to the RPE (Fig. 2H).

The immunoblots of the retina show the expression of aldolase C in the retina but not in the RPE, and it may be likely that other isoforms are present in the RPE. If other isoforms were expressed in the RPE, measuring aldolase activity would provide an unequivocal answer. We have standardized the aldolase activity assay for the retina and RPE using a coupled enzyme assay [23]. A decrease in absorbance at 340 nm was recorded as the measure of enzyme activity (Fig. 2I). Our assay results show that the retina has the highest aldolase activity, and RPE has very little aldolase activity (Fig. 2J). These observations suggest that aldolase expression may be absent in the RPE.

**Figure 3.**
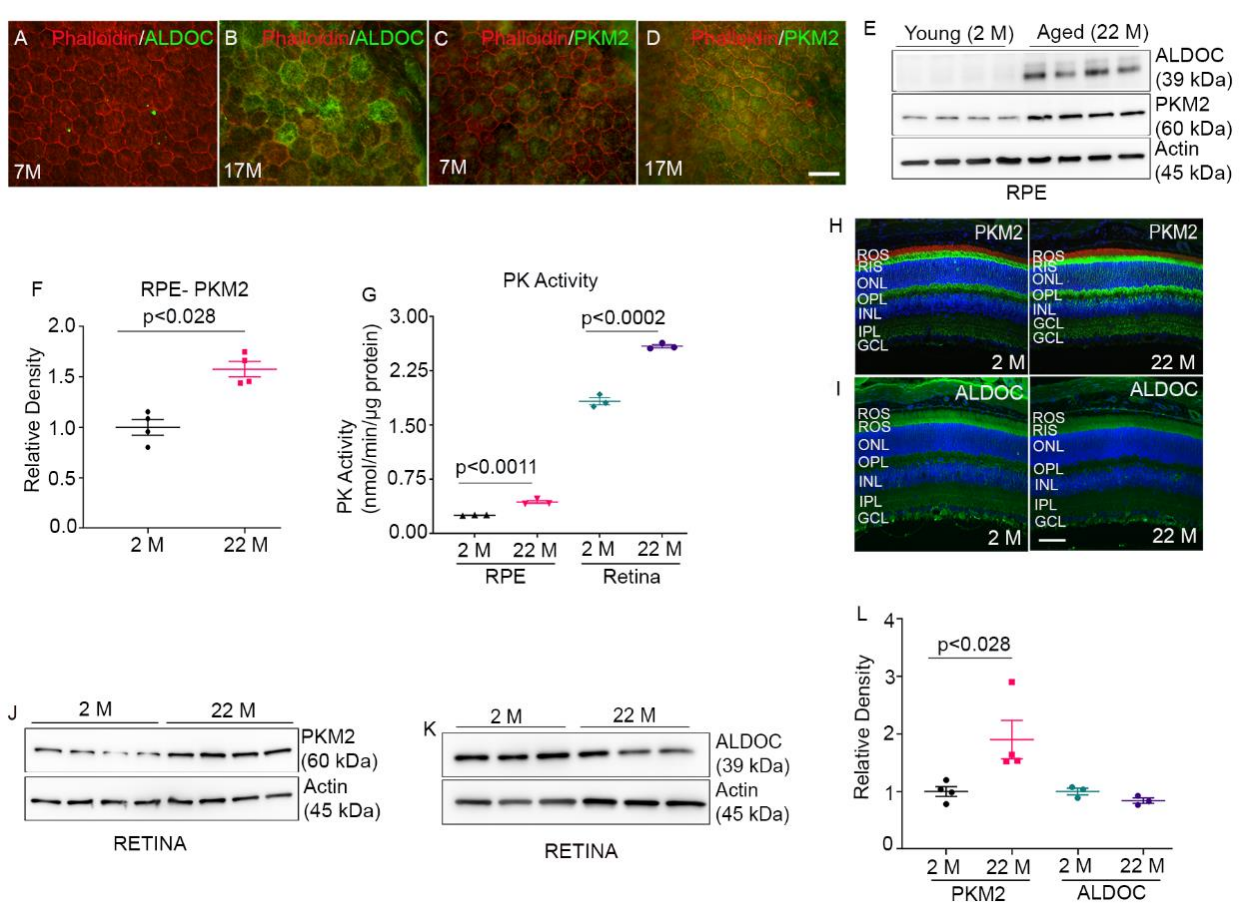
Expression of PKM2 and ALDOC in Young and Aged RPE. RPE flat mounts from 7-month-old (A, C) and 17-month-old (B, D) C57Bl6 mice stained with phalloidin (red), ALDOC (green in A and B), and PKM2 (green in C and D). Scale bar = 50 µm. Immunoblot analysis of RPE proteins from young (2M) and aged (22 M) mice with antibodies against ALDOC, PKM2, and actin (E). Densitometric analysis of PKM2 expression in 2- and 22-month-old RPE normalized to actin (F). Data (22 M expression relative to 2M) are mean ± *SEM (n=4).* Pyruvate kinase activity was measured in young and aged RPE and retina (G). Data are mean *+ SEM (n=3).* Immunostaining of young and aged mouse retina sections for PKM2 (H) and ALDOC (I). ROS, rod outer segments; RIS, rod inner segments; ONL, outer nuclear layer; OPL, outer plexiform layer; INL, inner nuclear layer; IPL, inner plexiform layer; GCL, ganglion cell layer. Scale bar=50 μm. Immunoblot analysis of retina proteins from 2M and 22 M mice with antibodies against PKM2 (J), ALDOC (K), and actin. Densitometric analysis of tested proteins normalized to actin (L). Data are mean + *SEM (n=4* for PKM2 and *n=3* for ALDOC). We used the nonparametric Mann-Whitney U test to examine the significance between the two groups.

**Figure 4.**
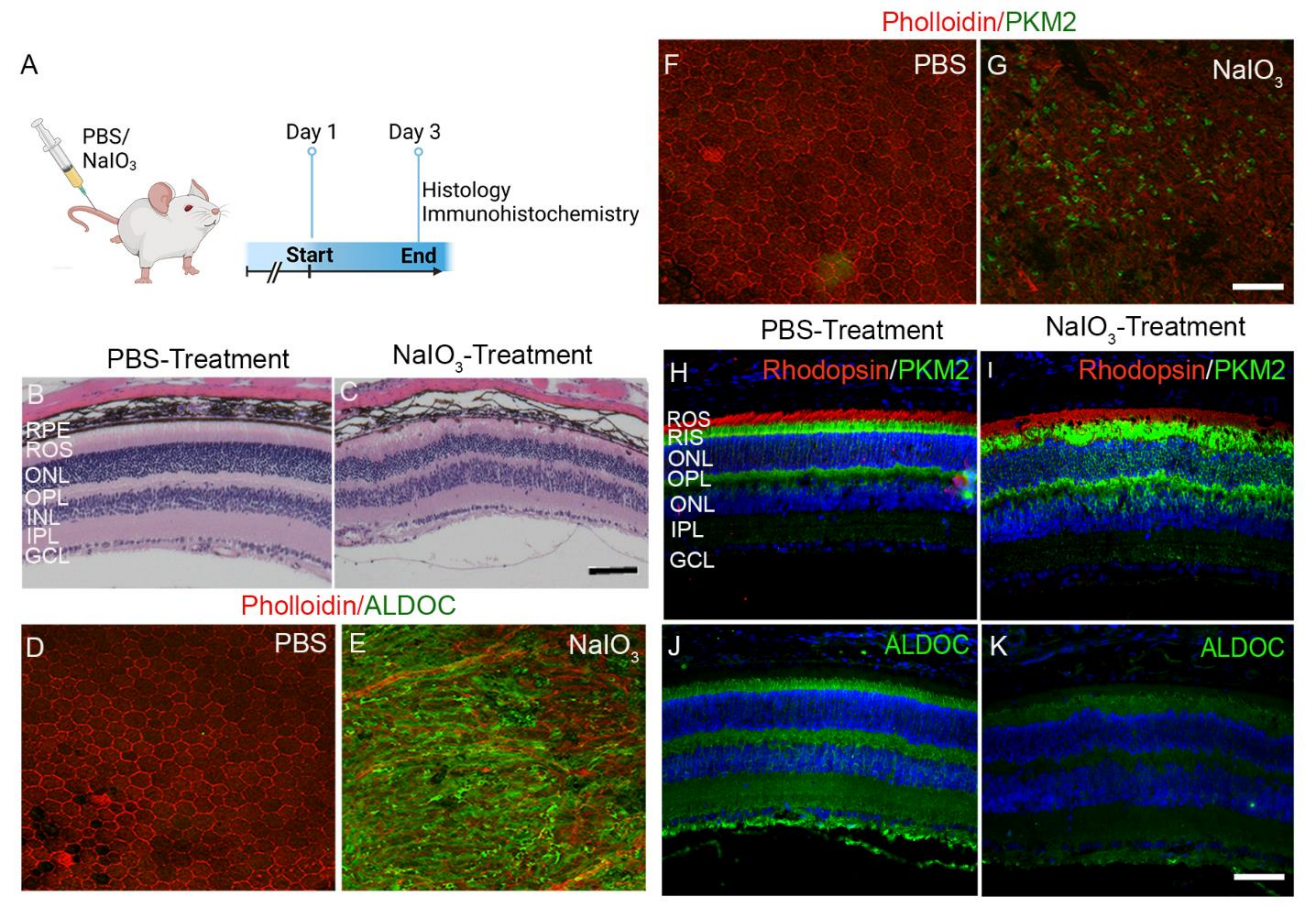
Effect of sodium iodate (NaIO3) on the expression of ALDOC and PKM2 in the RPE and retina. C57Bl/6 mice were tail vein injected with either PBS or NaIO_3_ (20 mg/kg) and 3 days later mice were examined for histology and immunohistochemistry (A). Retina morphology of PBS-treated (B) and NaIO3-treated (C) mice was examined using H & E staining. RPE flat mounts were prepared from PBS and NaIO3-treated mice and stained with ALDOC (D, E) and PKM2 (F, G) and counterstained with phalloidin. PBS and NaIO3-treated retina sections were stained for rhodopsin (H, I), PKM2 (H, I), and ALDOC (J, K) antibodies. ROS, rod outer segments; RIS, rod inner segments; ONL, outer nuclear layer; OPL, outer plexiform layer; INL, inner nuclear layer; IPL, inner plexiform layer; GCL, ganglion cell layer. Scale bar, 50 μm.

### Increased expression of ALDOC and PKM2 in aged-RPE and Retina

RPE flat mounts from 7-month-old and 17-month-old C57Bl6 mice were stained with ALDOC and PKM2 antibodies. The results indicate increased expression of ALDOC and PKM2 in aged RPE (Fig. 3B, D) compared to younger RPE (Fig. 3A, C). We immunoblotted RPE proteins from 2-month-old and 22-month-old mice with ALDOC and PKM2 antibodies. Our results indicate increased levels of ALDOC and PKM2 in the RPE of 22-month-old mice compared to those of 2-month-old mice (Fig. 3E). The quantification data indicate that PKM2 expression in the RPE is significantly higher in aged RPE compared to younger RPE (Fig. 3F). To determine the effect of increased expression of PKM2 on pyruvate kinase activity, we measured PK activity in young and aged-RPE samples. The results indicate that aged RPE shows a significantly higher PK activity compared to younger RPE (Fig. 3G). These observations suggest that aged RPE cells could be more glycolytic.

Retina sections from 2-month and 22-month-old mice were stained with PKM2 and ALDOC antibodies and the results indicated increased expression of PKM2 and decreased expression of ALDOC in 22-month-old mouse retina compared to 2-month-old mouse retina (Fig. 3H, I). Immunoblot analysis further confirms immune-histochemistry that a 22-month-old retina has significantly increased levels of PKM2 and decreased levels of ALDOC compared to a 2-month-old mouse retina (Fig. 3J-L). We measured PK activity in the 2-month and 22-month-old retina, and the results indicate that the aged retina shows a significantly increased pyruvate kinase activity compared to the young retina (Fig. 3G). These observations collectively suggest that aging altered the expression of PKM2 and ALDOC in the RPE and retina.

### Effect of NaIO_3_ on PKM2 and ALDOC expression in the RPE and retina

Sodium iodate-induced retinal degeneration represents a pre-clinical model of AMD and the features are similar to AMD characteristics of RPE dystrophy and geographic atrophy [16]. Sodium iodate is a known retina toxin that specifically damages RPE, which leads to a secondary effect on photoreceptor cells [24]. Tail vein injection of either PBS or NaIO_3_ to mice for 3 days followed by histological analysis indicated a marked morphological change in the retina and RPE of NaIO_3-_treated mice compared PBS injected mice (Fig. 4A). The results indicate marked RPE damage and disorganization of the neurosensory retina, especially rod outer and inner segments (Fig. 4C) and the retina integrity was well preserved in PBS-treated mouse retina (Fig. 4B). RPE flat-mount were prepared from PBS and NaIO_3-_treated mice, stained with ALDOC and PKM2 antibodies and counterstained with phalloidin. The results indicate that there was no staining of ALDOC and PKM2 expression in the RPE flat mounts in PBS-treated mice whereas the expression of ALDOC and PKM2 were higher in RPE flat mounts of NaIO_3_-treated mice compared to PBS-treated mice (Fig.4 D-G). The phalloidin staining is well decorated with the hexagonal RPE cell in PBS-treated mice (Fig. 4D, F) whereas RPE is disorganized in NaIO_3_-treated mice as evident from the irregular phalloidin staining (Fig. 4E, G). Prefer-fixed retina sections prepared from PBS-treated and NaIO_3_-treated mice were stained with rhodopsin, PKM2, and ALDOC antibodies. The rhodopsin expression was uniform in the rod outer segments (ROS) of PBS-treated mice whereas, the rhodopsin staining was not uniform in the NaIO_3_-treated mice and the ROS layer is disorganized (Fig. 4H, I). The expression of PKM2 is found specifically in rod inner segments and the outer plexiform layer in PBS-treated mice (Fig. 4H). However, in NaIO3-treated mice, PKM2 localization loses its boundary and can be observed in the outer nuclear layer and inner retina (Fig. 4I). ALDOC expression is found in rod inner segments, outer plexiform layer, inner plexiform layer, and ganglion cell layer in mice treated with PBS. However, in NaIO3-treated mice, the expression of ALDOC is reduced in these layers (Fig. 4J, K). These findings indicate that in AMD, both PKM2 and ALDOC expression is significantly altered in the RPE and neural retina.

## DISCUSSION

By the age of 65, one out of every three Americans experiences some form of vision-impairing eye condition. Among elderly individuals, one of the four major eye-related diseases—macular degeneration, diabetic retinopathy, glaucoma, and cataracts, collectively known as age-related eye diseases (AREDs)—typically affects them [25]. Earlier studies indicated that individuals with Wet AMD showed immunoreactivity to retinal antigens, particularly retinol-binding protein 3 and ALDOC, in serum IgG [7]. PKM2, on the other hand, was targeted by both dry and wet AMD patient sera [7]. The research demonstrated that the levels of anti-PKM2-IgG antibodies correlated most strongly with the stage of AMD [7], suggesting the potential involvement of autoimmunity in AMD pathogenesis. Additionally, changes in the expression of PKM2 and ALDOC were observed in the retina and RPE of aged mice [7].

Hurley’s research group on retinal metabolism found minimal age impact with impaired glutamine anaplerosis in eyecups [26]. The stability of these metabolic processes *ex vivo* suggests that age-related changes may not be inherent. Further experiments and needed to explore whether nutrient supply, oxygen availability, or structural changes influence retinal metabolism *in vivo* [26]. The retina and RPE rely on each other for survival and optimal functioning due to their complementary metabolic roles. This interdependence is crucial for their abilities to carry out essential and specialized functions [2]. Consistent with this symbiotic relationship, in mice, altered photoreceptor metabolism causes late-stage age-related macular degeneration-like pathologies [27]. Earlier studies show that photoreceptors are glycolytic whereas RPE cells are dependent on OXPHOS working through a metabolic ecosystem [5].

Interestingly, aged RPE switches from OXPHOS to glycolysis [28]. We have not examined the expression of glucose (GLUTs) and lactate (MCT) transporters in this study. Our previous studies show that ablation of PKM2 in photoreceptor cells results in decreased expression of GLUT1 [29]. Nevertheless, prior research emphasizes that the metabolic activity of the retina and RPE diminishes as individuals age [30-32], and stress renders mitochondria more susceptible to oxidative stress [33]. Dysfunction of mitochondria in the RPE can decelerate β-oxidation and lead to the accumulation of lipids [34], compelling the RPE to increase its utilization of glucose [5]. AMD exacerbates the alterations in metabolism linked to the aging process [35-37]. The increased PKM2 expression and increased pyruvate kinase activity suggest an increased glycolysis in the RPE. An overactivation of mTOR has been identified in RPE cells obtained from aged donors [38], indicating a correlation between aging and metabolic stress. Previous studies indicate that activating PKM2 during acute outer retina stress with small molecule inhibitors promotes the survival of photoreceptors [39]. Conversely, in the retinitis pigmentosa (RP) mouse model, the removal of PKM2 partially rescues the degenerative phenotype (76). These findings suggest that reducing photoreceptor anabolism in RP seems somewhat beneficial while activating PKM2 in stress conditions proves advantageous. Beyond its role as a glycolytic enzyme, PKM2 also serves as a transcriptional co-activator in the regulation of gene expression [3]. When phosphorylated, PKM2 translocates into the nucleus, activating the transcription of β-catenin and signal transducer and activator of transcription 5 (STAT5) [40]. This activation facilitates the expression of various genes, including cyclin D1, LDHA, PKM2, GLUT1, and cMyc [40]. This process redirects glucose metabolism, which is crucial for cancer progression [40]. In our current study, we observed an increased expression of PKM2 in aged RPE. This finding aligns with a published study on AMD, suggesting a role for PKM2 in transcriptional gene regulation in the RPE. Reports suggest that mitochondrial dysfunction in the RPE can hinder β-oxidation, leading to lipid accumulation [34].

Consequently, the RPE increases its reliance on glucose utilization [5]. Further gene expression studies in aging and AMD are necessary to delineate the specific roles of elevated PKM2 in RPE metabolism and transcription.

Interestingly, as RPE cells age, there is a notable shift from OXPHOS to glycolysis [28]. These aging cells display disorganized mitochondrial membranes, loss of cristae, and the accumulation of lipofuscin [41]. The heightened oxidative stress in these aged RPE cells increases their susceptibility to dysfunction, cellular senescence, and cell death, contributing to the aging process and age-related diseases such as AMD [41]. The loss of RPE differentiation is a recognized factor in various retinal diseases, including inherited rod-cone degenerations, inherited macular degeneration, AMD, and proliferative vitreoretinopathy [42]. In these instances, RPE cells undergo a crucial epithelial-mesenchymal transition (EMT) and express Transforming Growth Factor Beta (TGFβ) [42]. TGFβ2-induced EMT in RPE suppresses the expression of peroxisome proliferator-activated receptor-gamma coactivator-1 alpha (PGC-1α), leading to compromised mitochondrial integrity, reduced expression of genes related to mitochondrial dynamics, and decreased mitochondrial OXPHOS levels [43]. These mitochondrial changes result in a compensatory increase in glycolysis [43]. Notably, the application of ZLN005, a PGC-1α activator, effectively counteracts TGFβ2-induced EMT, suggesting its potential as a therapeutic approach for subretinal fibrosis. These findings underscore the interconnected relationship between EMT, mitochondrial dysfunction, and metabolic shifts in RPE cells [43].

Sodium iodate (NaIO3)-induced retinal degeneration is a widely used model to investigate late-stage dry AMD [16]. Our current study shows that both aged RPE and NaIO_3_-induced AMD RPE show increased expression of PKM2 and ALDOC in the RPE whereas ALDOC expression is decreased in neural retina. Several proteomic analysis studies on oxidative light damage [44], human RPE cells in culture, and human donor eyes [45] identified ALDOC as one of the target proteins nonenzymatically oxidized by reactive aldehyde 4-hydroxynonenal (4-HNE). Further studies are needed to investigate whether ALDOC undergoes oxidation by 4-HNE in aged conditions, potentially leading to an extended half-life of the protein in the RPE.

Alodlase C has been shown to positively regulate the canonical Wnt signaling pathway [46]. Wnt signaling may be disturbed in AMD patients, which could contribute to the retinal inflammation and increased A2E levels found in AMD. The inhibition of ALDOC reduced lactate production in colorectal cancer cells [47]. In gallbladder cancer, ALDOC knockdown significantly downregulated glucose uptake, glycolysis, and cell proliferation [48].

The ALDOC is predominately expressed in the retina and colocalized with PKM2 in the photoreceptor cells, suggesting the FBP activity might be tightly regulated by ALDOC for PKM2-mediated Warburg effect. ALDOC was not expressed in the younger RPE. However, in aged mice, ALDOC expression was observed. In the retina, the ALDOC is significantly reduced. ALDOC cleaves FBP to generate 3-carbon sugars, especially, the DHAP which is important for the synthesis of glycerol, which is a backbone for phospholipids [12]. In the lipid bilayer, one molecule of rhodopsin requires sixty molecules of phospholipids, suggesting a high rate of glycerol/phospholipid synthesis in photoreceptors [49]. Every day, by the onset of light, 10% of photoreceptors are phagocytosed by the RPE [50], and the photoreceptor cells have a high demand for synthesizing lipids, nucleic acids, the NADPH to maintain the length of the photoreceptor cells for retina health and maintenance. Aerobic glycolysis is needed to perform these functions, and the predominant expression of ALDOC in the retina suggests that the Warburg effect is sustained by the hydrolysis of FBP to inactivate the PKM2 to redirect glucose for anabolic processes. Besides, the presence of ALDOC in the retina and the absence of this enzyme in the RPE suggest that the synthesized lipid could be transported to RPE similar to the metabolic ecosystem described for glucose and lactate transport between photoreceptor cells and RPE [5].

PKM2 is predominantly expressed in the photoreceptor cells and weak expression of PKM2 in the RPE. Reduced pyruvate kinase (PK) activity supports the Warburg effect and increased PK activity supports OXPHOS. In aged-RPE, PKM2 expression was higher, and the PK activity was significantly higher compared to younger RPE. These observations suggest that RPE is glycolytic similar to photoreceptor cells.

**Figure 5.**
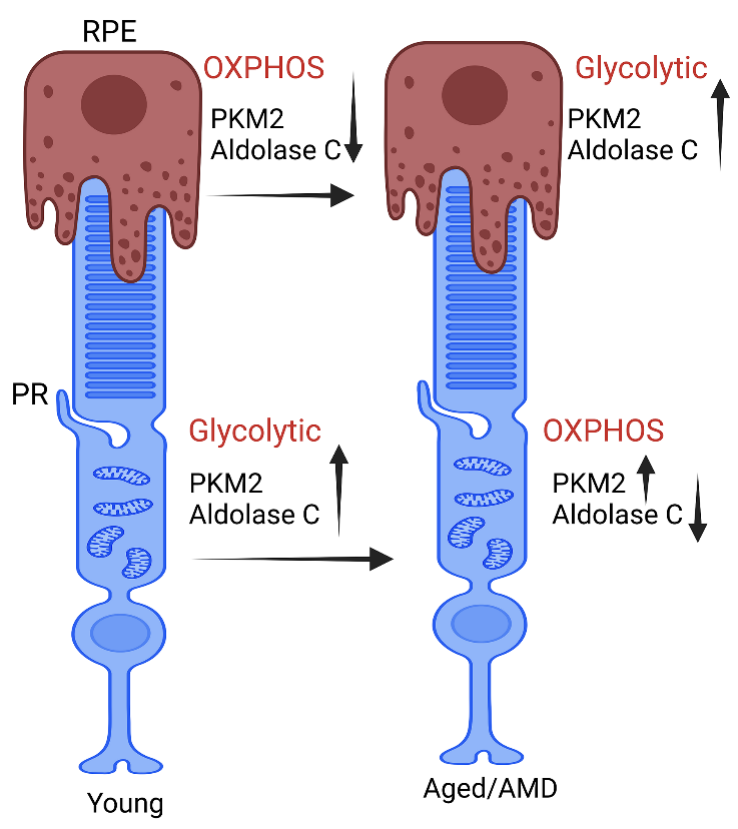
The image depicts metabolic alterations in the glycolytic enzymes ALDOC and PKM2 during aging and AMD. Our research indicates an increase in the expression of PKM2 and ALDOC in the RPE of aged and AMD mice. Conversely, we observed a decrease in ALDOC expression but an increase in PKM2 expression in the retina of aged and AMD mice. This study emphasizes that aged and AMD RPE cells tend to prefer aerobic glycolysis (switching OXPHOS to glycolytic), while this tendency is diminished in the aged and AMD retina (decreased aerobic glycolysis and switching glycolytic to OXPHOS). RPE, retinal pigment epithelium; PR, photoreceptor cell; ALDOC, aldolase C; PKM2, M2 isoform of pyruvate kinase; AMD, age-related macular degeneration; OXPHOS, oxidative phosphorylation.

Ideally, all experiments involving aged mice would be conducted on 22-month-old individuals. However, due to potential mortality before reaching this age, we acknowledge that some mice may not survive until this time point. Additionally, our experience suggests that preparing RPE-flat mounts from aged mice can be challenging, occasionally resulting in the inability to isolate intact RPE. Consequently, we opted to prepare and examine flat mounts at 17 months, while conducting all biochemical analyses on 22-month-old mice. The experiments described in this manuscript primarily cover the older age range of 17-24 months [51]. Notably, the expression of PKM2 and ALDOC in 17-month-old RPE correlated well with the protein expression observed in 22-month-old RPE on immunoblots, and the same held true for the younger age group, between 2 and 7 months.

**Table 1:** Phenotypes in healthy photoreceptors, healthy RPE, Aged and AMD RPE, and Aged AMD photoreceptor cells.

Healthy RPE	Aged and AMD RPE
•Oxidative phosphorylation •Decreased Lipid synthesis	•Increased Glycolysis •Increased lipid synthesis• •Decreased NADPH production •Oxidative stress
Healthy Photoreceptor cells	Aged and AMD Photoreceptor cells
•Increased Glucose transport from RPE •Increased Glycolysis •Increased Lipid synthesis •Increased Lactate production •Increased NADPH production	•Reduced glucose transport from RPE •Increased Oxidative phosphorylation •Reduced glycolysis •Reduced Lipid synthesis •Reduced NADPH synthesis •Reduced Lactate production •Oxidative stress

The increased ALDOC expression in the aged-RPE suggests an increased phospholipid synthesis, decreased ALDOC expression in the retina suggests a reduced phospholipid synthesis, and reduced ALDOC expression decreases the breakdown of FBP that may bind to PKM2 and promote OXPHOS. Consistent with this idea, the PK activity in the aged retina is significantly higher compared to the younger retina, despite increased expression of PKM2 in the aged retina. Combined data on ALDOC and PKM2 in retina and RPE suggest that in aged RPE, the cells may utilize glucose for glycolysis instead of sending it to the retina and the RPE may synthesize lipids instead of depending on photoreceptor cells. We observed the aged-RPE phenotype of ALDOC and PKM2 overexpression in mice injected with retina toxin, NaIO_3_, a pre-clinical mouse model of AMD. This altered ALDOC and PKM2 in these two cell types may contribute to the pathogenesis of AMD (Fig. 5). Further studies are needed to understand the lipid synthesis and transport of lipids from photoreceptor cells to RPE and *vice versa* in health and disease.

This study draws some important conclusions and opens questions (Table 1). Based on the earlier studies and the data from the current study suggest that healthy RPE has increased OXPHOS and decreased lipid synthesis whereas in aged and AMD-RPE, increased glycolysis, increased lipid synthesis, decreased NADPH production, and increased oxidative stress. In young photoreceptor cells, increased glucose transport from the RPE, increased glycolysis, increased lipid synthesis, increased lactate production, and increased NADPH production whereas in aged and AMD photoreceptors, the cells may have reduced glucose transport from the RPE, increased OXPHOS, reduced glycolysis, reduced lipid synthesis, reduced NADPH production, reduced lactate production and increased oxidative stress.
